# Seed priming with selenium and zinc nanoparticles modifies germination, growth, and yield of direct-seeded rice (*Oryza sativa* L.)

**DOI:** 10.1038/s41598-022-11307-4

**Published:** 2022-05-02

**Authors:** Saju Adhikary, Benukar Biswas, Debashis Chakraborty, Jagadish Timsina, Srikumar Pal, Jagadish Chandra Tarafdar, Saon Banerjee, Akbar Hossain, Sovan Roy

**Affiliations:** 1 Department of Agronomy, Bidhan Chandra Krishi Viswavidyalaya, Mohanpur, Nadia, West Bengal 741 252 India; 2 Division of Agricultural Physics, Indian Agricultural Research Institute, New Delhi, 110 012 India; 3 Global Evergreening Alliance, 1 Vision Drive, East Burwood, Melbourne, VIC 3151 Australia; 4 Institute for Study and Development Worldwide, Sydney, NSW Australia; 5 Department of Agricultural Biochemistry, Bidhan Chandra Krishi Viswavidyalaya, Mohanpur, Nadia, West Bengal 741 252 India; 6 Ex-Scientist, Central Arid Zone Research Institute, 17E/361A, C. H. B., Jodhpur, 342008 India; 7 32/E/2/1, BanamalipurBarasat, Kolkata, West Bengal 700124 India; 8 Department of Agricultural Physics and Meteorology, Bidhan Chandra Krishi Viswavidyalaya, Mohanpur, Nadia, West Bengal 741 252 India; 9 Bangladesh Wheat and Maize Research Institute, Dinajpur, 5200 Bangladesh; 10 Department of Science & Technology and Biotechnology, Vigyan Chetana Bhavan, Kolkata, West Bengal 700064 India

**Keywords:** Biological techniques, Plant sciences, Environmental sciences

## Abstract

Direct-seeded rice (DSR) seeds are often exposed to multiple environmental stresses in the field, leading to poor emergence, growth and productivity. Appropriate seed priming agents may help to overcome these challenges by ensuring uniform seed germination, and better seedling stand establishment. To examine the effectiveness of sodium selenite (Na-selenite), sodium selenate (Na-selenate), zinc oxide nanoparticles (ZnO-NPs), and their combinations as priming agents for DSR seeds, a controlled pot experiment followed by a field experiment over two consecutive years was conducted on a sandy clay loam soil (*Inceptisol*) in West Bengal, India. Priming with combinations of all priming agents had advantages over the hydro-priming treatment (control). All the combinations of the three priming agents resulted in the early emergence of seedlings with improved vigour. In the field experiment, all the combinations increased the plant chlorophyll, phenol and protein contents, leaf area index and duration, crop growth rate, uptake of nutrients (N, P, K, B, Zn and Si), and yield of DSR over the control. Our findings suggest that seed priming with the combination of ZnO-NPs, Na-selenite, and Na-selenate could be a viable option for the risk mitigation in DSR.

## Introduction

Rice plays a major role in achieving global food security but the crop now is under several threats, including yield stagnation in major rice-producing regions, negative environmental impacts of the overuse of irrigation water and agrochemicals, high labour requirements which is under short supply, and increasing concerns of loss of natural habitats due to intensive rice cultivation^1–3^. In particular, traditional puddled-transplanted rice (PTR) requires more irrigation water and labour than direct-seeded rice (DSR)^4^. Future, production systems should be more productive, cost-effective, and remunerative while minimizing the negative environmental impacts. The DSR could potentially be a viable alternative to PTR due to reduced labour requirement, increased resource (water, nutrient, energy, etc.) use efficiency, and higher returns^5,6^. Direct seeding can be achieved by sowing on the dry soil either mechanically or manually (Dry DSR), on wet soil through broadcasting (Wet DSR), or in standing water through broadcasting (Water seeding)^5,7^. Dry DSR could be more advantageous over the other two methods^8,9^ due to less time required for sowing, thus economizing time and labour, and saving water. Though PTR is the main rice transplanting system in Asia including India, DSR has now been gaining popularity^10–13^. For example, in China, DSR now covers 28% of the total rice area^14^, while in Sri Lanka and Malaysia, areas under DSR are > 93% (1.03 M ha) and 95% (0.67 M ha) respectively of their respective total rice area^15^. In the Indian Punjab alone, DSR was adopted in 0.601 M ha in 2021^16^.

Rapid loss of seed germination and vigor during storage and poor germination and seedling stand establishment in the field is identified as the major factors for the low productivity of DSR in South Asia^14,17–20^. In DSR when seeds are sown directly in the field, plants are often exposed to multiple environmental stresses, particularly during emergence and early development^21–23^. These stresses depress the kinetics of many physiological and metabolic processes^24^ and generate a large number of reactive oxygen species (ROS) in plant cells that trigger lipid peroxidation in membranes^25–27^. These are followed by damage to biomolecules including proteins, carbohydrates, and DNA^26,28^, and reduce respiration rate and energy supply to growing plant tissue^29^. All these processes affect sequentially the germination and seedling establishment, plant stand, growth and resource utilisation, and ultimately the yield of DSR.

The conventional breeding approach to develop varieties for enhanced germination and plant establishment is time-consuming and genetic engineering is also highly controversial albeit with its high potential benefits^30^. To overcome such difficulties, a large number of abiotic stress-tolerant rice genes have been characterized during the last three decades which are being further exploited for varietal development with higher productivity^31,32^. In addition to the above methods which are generally costly and time-consuming, seed priming with various priming agents has proven utility in optimising seed vigour and plant physiological processes, which make the plant ready to face multiple stresses in the field more efficiently^33–35^. Several successful priming agents have been reported in the literature ranging from salts, polyamines, hormones, compatible solutes, and aqueous plant extracts^36^.

Zinc (Zn), an essential micronutrient, is the only metal that forms a part of the six different classes of enzymes^37^. It is closely involved in many biochemical and physiological processes^38^. Seed priming with Zn has shown positive effects on seed vigour, germination, early seedling growth and biomass production, photosynthetic efficiency, and increasing the contents of sugar, total nitrogen, protein, and micronutrients in many crops^39–41^. In the recent past, biologically synthesised zinc oxide nanoparticles (ZnO-NPs) with their enhanced physical and biochemical characteristics and low environmental toxicity have shown their efficacy as priming agents^42,43^. Literature indicates that ZnO-NPs have beneficial effects at low concentrations (about 50 ppm) but detrimental effects at concentrations above 500 ppm on plant growth and development^44^. Recently, extensive work is going on the use of ZnO-NPs in nanooncology and mitigation of its associated toxicity risk on lungs, liver, kidney, pancreas, spleen, stomach, testis, thymus, brain, heart, blood, etc^45^. Uniformity in seedling emergence and improved growth of rice plants using ZnO-NPs as priming agent was also reported with higher production of antioxidant enzymes against ROS damage^46,47^.

In plants, selenium (Se) as a constituent of seleno-proteins, has been reported to enhance starch and ATP synthesis^48^, regulate water status, prevent chlorophyll loss during drought^49,50^, and delay senescence^51^. In addition, it regulates redox reactions^52,53^ and ROS concentration, and consequently lipid peroxidation^54^. In rice, priming with Na-selenite has been shown to trigger its seed germination^55^. Subsequent studies also reported the efficacy of rice seed priming with Na-selenate^56^ and selenite-selenate combination of sodium salt^57^ with their contributions to the assimilation and storage pathway respectively. Oxyanions of Se can promote the adsorption of Zn and enhance their bioavailability within the plant system by synergy^58,59^. To our knowledge, there is no literature on the combined application of zinc or nano-zinc with Se as seed priming agents on germination and seedling establishment and growth and yield of DSR.

In response to the above gaps, we hypothesized that (1) the (synergistic) effect of ZnO-NPs and Se as seed priming agents in DSR cultivation influences seed germination, and the emergence of healthy and robust seedlings capable of mitigating environmental stresses; and (2) the synergistic effect could result in higher DSR yield compared to that without the seed priming. To test these hypotheses, a controlled pot experiment and a field experiment were undertaken with different treatments of ZnO-NPs, Na-selenate, and Na-selenite either singly or in their combinations as priming agents in DSR.

## Materials and methods

### Experimental site

A pot experiment (examined for seed vigour) was conducted followed by field experimentation (examined for plant growth traits and yield) each year (2019 and 2020) on DSR at Central Research Farm of Bidhan Chandra Krishi Viswavidyalaya, West Bengal, India (23° 5.3ʹ N, 83° 5.3ʹ E; subtropical; 9.75 m MSL). The soil was sandy clay loam (sand 64.8%, silt 10.4%, and clay 24.8%) with EC of 0.296 dSm^−1^ and a pH of 7.3^60^. Initial soil properties of the study site were: 11.2 g kg^−1^ oxidizable organic carbon^61^, 315 kg ha^−1^ available N^62^, 41.6 kg ha^−1^ available P^63^, and 156.4 kg ha^-1^ available K^64^. The climate is tropical moist sub-humid, with hot summer, and moderately cold winter. Average maximum and minimum temperatures ranged from 25 to 36 °C in summer and from 10 to 25 °C in winter (Supplementary Figure S1).

### Experimental treatments

ZnO-NPs used in this study were with mean hydrodynamic diameter of size distribution (determined by dynamic light scattering technique) below 10 nm, surface charge (measured as zeta potential) of − 5.7 mV and maximum intensity at 1 keV (TEM–EDX) and these biologically synthesized nanoparticles were stable up to 90 days in the aqueous medium^65^. Eight treatment combinations of ZnO-NPs and Se were selected: T_1_: Hydropriming with distilled water (Control); T_2_: Na-selenite at 50 µmol (as Na_2_SeO_3_;Sigma-Aldrich USA); T_3_: Na-selenate at 50 µmol (as Na_2_SeO_4_;Sigma-Aldrich USA); T_4_: Na-selenite at 50 µmol + Na-selenate at 50 µmol; T_5_: ZnO-NPs at 10 µmol ; T_6_: ZnO-NPs at 10 µmol + Na-selenite at 50 µmol; T_7_: ZnO-NPs at 10 µmol + Na-selenate at 50 µmol; and T_8_: ZnO-NPs at 10 µmol + Na-selenite at 50 µmol + Na-selenate at 50 µmol]. Indica inbred semi-dwarf type medium slender grain early maturing (108–110 d) rice variety Ajit IET 22066^66^ was used in both pot and field experiments.

### Pot experiment

The pot experiment was conducted using a completely randomized design in three replications with factorial arrangements. Twenty-five g of seeds were placed in a 200 ml conical flask containing 125 ml initiator solution as per the priming agent treatments, and aerated distilled water for the control. Seeds were kept for imbibitions for 24 h. The flasks were placed in an incubator [darkness, 27 ± 3 °C and 80% relative humidity] and were shaken once every 6 h. After priming, the seeds were filtered, placed in distilled water for 20 min, and rinsed five times with ultrapure water. Autoclaved glass Petri dishes were lined with double layers of filter paper and were placed on a laboratory bench for air drying for a period of 24 h. Thereafter, 10 ml sterile water was aseptically pipetted into and 100 seeds were placed in each Petri-dish. Each seed soaking treatment was performed in triplicate Petri-dishes, which were kept inside an incubator at 26 ± 0.5 °C under aseptic conditions.

Seeds were sown in plastic pots (15.6 cm height, 18.2 cm top diameter, and 12.5 cm lower diameter), which were filled with air-dried and well-mixed field soil (2000 g) collected from the study site. Seeds were sown uniformly in each pot with soil moisture at field capacity (−  0.03 MPa). Pots were placed in a screen house under the natural condition with a 14/10 h light/dark photoperiod and uniformly irrigated with distilled water as per the requirement to avoid water deficit. The seed emergence was counted daily according to the Association of Official Seed Analysts (AOSA)^67^ until a constant count was achieved. A seed was considered as “emerged” when the hypocotyl length was ≥ 2 mm. Time taken to 50% emergence of seedlings (E_50_) was calculated according to the modified formula of Basra et al.^68^:

1 $${E}_{50}=\mathrm{ti}+\frac{\left(\frac{\mathrm{N}}{2}-\mathrm{ ni}\right)(\mathrm{tj }-\mathrm{ ti})}{(\mathrm{nj }-\mathrm{ ni})}$$

where N is the final number of emerged seeds; ni and nj are the cumulative number of seeds emerged by adjacent counts at times ti and tj where ni < N/2 < nj. Mean emergence time (MET), an indicator of the relative emergence of seedlings in a day was calculated according to Ellis and Roberts^69^:

2 $$\mathrm{MET}= \frac{\Sigma (\mathrm{D}*\mathrm{ n})}{\mathrm{ \Sigma n}}$$

where n is the number of new emerging seeds on day D (number of days from the beginning of emergence). The emergence index (EI), which is a measure of the percentage and rate of germination, was calculated as described by AOSA^70^:

3 $$\mathrm{EI}=\frac{\text{Number: of emerged seeds}}{\text{Days of the first count}}+\dots + \frac{\text{Number of emerged seeds}}{\text{Days of the final count}}$$

Vigour index (VI) was calculated after Wang et al.^71^.

4 $$\mathrm{VI}=\mathrm{Sd }\times \sum \left(\frac{\mathrm{Gt}}{\mathrm{t}}\right)$$

where Sd is the seedling's dry weight at the end of the test period (7 days), Gt is the number of germinated seeds on day t from the beginning of the test.

Root and shoot lengths were measured 18 days after sowing (DAS) from each treatment. For these, five randomly selected seedlings were oven-dried at 70 °C for 48 h to get the dry biomass of root and shoot, and both the components were summed up to record the total seedling biomass.

### Field experiment

The experiment was laid out in a factorial randomized complete block design with three replications. Fields were prepared by cultivating twice using a disc harrow (Unison Exports, Ludhiana, Punjab, India), followed by levelling with a wooden board. Pre-germinated primed seeds were sown manually by a single-row planter having an inclined plane seed metering mechanism at 25 kg ha^−1^ at 20 cm row-to-row spacing. The field was surface-irrigated immediately after sowing. Soil water potential was monitored with tensiometers installed at 20 cm depth, and the field was irrigated at − 30 kPa potential. Fertilizers were applied as per soil test-based recommendations^72^ with a basal application of N at 25 kg ha^−1^, P_2_O_5_ at 50 kg ha^−1^, K_2_O at 50 kg ha^−1^, and ZnSO_4_ at 5 kg ha^−1^ as urea, single super phosphate and muriate of potash, respectively. Additionally, 75 kg N ha^−1^ in the form of urea and ammonium sulphate (AS) was applied in two splits: one half at the active tillering stage (urea and AS in equal proportion) and the other half at the panicle initiation stage (only through AS). Weeds were controlled by a pre-emergence herbicide (pendimethalin at 0.75 kg a.i. ha^−1^) at 2 DAS, followed by a post-emergence herbicide (bispyribac-sodium at 25 g a.i. ha^−1^) at 20 DAS. Weeds that escaped these treatments were removed manually at 42 DAS. Chloropyriphos at 50 g a.i. ha^−1^ and propiconazole at 62.5 g a.i. ha^−1^ was used to control insects and diseases, respectively. Irrigation was withdrawn 15 days before harvest. All agronomic management practices were the same in both years. Grains were harvested at 15–18% grain moisture content.

### Growth traits analysis

Plants were collected from 1-m length within a row in each plot at the initiation of tillering (14 DAS), panicle initiation (42 DAS), 50% flowering stage (70 DAS), and physiological maturity (108 DAS) to determine aboveground biomass and green leaf area index (LAI). Green leaves were separated and the area was measured with a leaf area meter (LI-3100, LI-COR, Lincoln, Nebraska, USA). The LAI was expressed as leaf area per unit of the area sampled for each plot. Dry weights of each plant part were determined after oven-drying at 80̊ C to calculate crop growth rate (CGR), net assimilation rate (NAR), and leaf area duration (LAD)^73^ for vegetative (up to 41 DAS), reproductive (42–69 DAS) and grain filling (70–108 DAS) stages using the following equations:

5 $$\mathrm{CGR}=\frac{{\mathrm{W}}_{2}-{\mathrm{W}}_{1}}{{\mathrm{t}}_{2}-{\mathrm{t}}_{1}}$$

6 $$\mathrm{NAR}=\frac{{\mathrm{W}}_{2}-{\mathrm{W}}_{1}}{{\mathrm{t}}_{2}-{\mathrm{t}}_{1}}\times \frac{{\mathrm{log}}_{\mathrm{e}}{\mathrm{L}}_{2}-{\mathrm{log}}_{\mathrm{e}}{\mathrm{L}}_{1}}{{\mathrm{L}}_{2}-{\mathrm{L}}_{1}}$$

7 $$\mathrm{LAD}=\frac{{\mathrm{L}}_{2}+{\mathrm{L}}_{1}}{2}\times \left({\mathrm{t}}_{2}-{\mathrm{t}}_{1}\right)$$

where W_1_ and W_2_ were dry weights of aerial plant parts per unit land area at time t_1_ and t_2_, respectively; L_1_ and L_2_ were total leaf area of plants per unit land area at time t_1_ and t_2_ respectively.

### Biochemical analysis of seedlings

Four random rice seedlings from each plot were collected on 18 DAS to determine total soluble phenol compounds^74^, photosynthetic pigments (chlorophyll “a” and “b”)^75^, and soluble proteins^76^.

### Nutrient uptake

Plant samples were collected at harvest, oven-dried, and ground. The N content on a dry weight basis was estimated by the Micro-Kjeldahl method^77^, P content by colorimeter method using vanadomolybdate yellow^77^, and K content by a flame photometer^78^. Zinc was analyzed through diacid extract using Perkin-Elmer Atomic Absorption Spectrophotometer. Boron was estimated by dry ashing and azomethine-H methods^79^, and silica was estimated by the blue silicomolybdic acid method^80^. The nutrient uptake by grain and straw was expressed on a per hectare basis^81^.

### Yield and yield components

Grain and straw yields were determined at physiological maturity. The harvest index was calculated as the ratio of dry grain yield to total dry biomass yield (both oven-dried at 70 °C). Tiller and panicle densities were determined with a quadrat (0.4 m × 0.5 m) placed randomly in each plot at two locations. At the same time, five plants were selected randomly from each plot to measure the number of filled grains panicle^−1^ and 1000-grain weight^82^.

### Statistical analyses

All data from pot and field experiments were analysed using a mixed ANOVA model in SAS, considering year and treatment, and their two-way interactions as factors. Treatment adjusted means were separated by using HSD Tukey multiple range test at 5% level of significance^83^. The coefficient of determination (R^2^) and correlogram was calculated to assess the degree of association between two variables using the JMP Pro 16.

## Results

### Pot experiment

#### Seed germination emergence and vigour, and enzymatic and biochemical activities

Seed priming improved gemination and vigour attributes, although year-to-year differences were evident (Supplementary Table S1). Priming with either Se or ZnO-NPs alone or in combination reduced the time to start of emergence (TSE), time to reach 50% germination (E_50_), and mean emergence time (MET) compared to the control, while EI and VI parameters improved over the control (Table 1). Percent reduction in TSE (43.3), MET (25.5), and E_50_ (30.9), and percent increase in EI (66.4) and VI (112.3) were greater with the combined application of ZnO-NPs, Na-selenate, and Na-selenite. Even Na-selenite and ZnO-NPs combination favoured the germination, as evidenced from the germination parameters except for TSE.

**Table 1:** Effect of seed priming on seedling emergence and seedling vigour of DSR in a pot experiment (pooled over 2019 and 2020).

Treatment	TSE (days)	E_50_ (days)	MET (days)	EI	VI
T_1_	3.8a	5.8a	6.19a	13.5f	969f
T_2_	3.2b	5.1bc	5.26bc	17.5e	1290e
T_3_	3.3b	4.9bc	5.06bc	20.1c	1519d
T_4_	3.3b	4.9bcd	5.15bc	17.6e	1590cd
T_5_	3.2b	4.7cd	5.49b	18.1de	1221e
T_6_	2.7c	4.7de	4.96bc	19.4cd	1643c
T_7_	2.7c	4.4e	4.72c	20.4bc	1796b
T_8_	2.2d	4.0f	4.61c	22.4a	2057a

*TSE* time to start emergence, *E*_*50*_ time taken to reach to 50% emergence, *MET* mean emergence time, *EI* emergence index, *VI* Vigour index.

*T*_*1*_ hydro priming or control, *T*_*2*_ Na-selenite at 50 µmol, *T*_*3*_ Na-selenate at 50 µmol, *T*_*4*_ Na-selenite at 50 µmol + Na-selenate at 50 µmol, *T*_*5*_ ZnO-NPs at 10 µmol, *T*_*6*_ ZnO-NPs at 10 µmol + Na-selenite at 50 µmol, *T*_*7*_ ZnO-NPs at 10 µmol + Na-selenate at 50 µmol, *T8* ZnO-NPs 10 µmol + Na-selenite at 50 µmol + Na-selenate at 50 µmol.

Means not sharing a letter in common differ significantly at 5% probability level by HSD Tukey’s test.

A minimum leachate conductivity of seeds was recorded in the combination of all three priming agents (Supplementary Fig. S2). Na-selenate was more effective in increasing antioxidant enzyme activities compared to Na-selenite, and the priming through the combination of Na-selenite, Na-selenate, and ZnO-NPs resulted in improved biochemical activities in DSR seeds (Supplementary Table S2).

### Field experiment

#### Chlorophyll, phenol, and soluble protein content in rice seedling

Total chlorophyll, phenol, and soluble protein contents in rice seedlings increased through priming of seeds over the control (Table 2). The combination of Na-selenite, Na-selenate, and ZnO-NPs recorded the highest total chlorophyll (5.39 mg g^−1^), phenol (0.58 mg g^−1^) and soluble protein (0.37 mg g^−1^) contents, although comparable with ZnO-NPs + Na-selenate treatment (chlorophyll, phenol and soluble protein were 5.27, 0.57 and 0.35 mg g^−1^).

**Table 2:** Effect of seed priming on total chlorophyll, phenolics, and soluble protein contents (mg g^-1^ fresh weight) in direct-seeded rice seedlings in a field experiment during 2019 (YI) and 2020 (YII).

Treatment	Total chlorophyll	Total phenolics	Soluble protein
**Year**			
YI	4.56n	0.55g	0.28k
YII	4.49o	0.54g	0.28k
**Treatment**			
T1	5.40h	0.50f	0.17j
T2	5.28h	0.52ef	0.25i
T3	4.68i	0.54def	0.30gh
T4	4.48j	0.56de	0.31gh
T5	4.26k	0.55de	0.25i
T6	4.17k	0.54def	0.26hi
T7	4.09l	0.56de	0.35fg
T8	3.82m	0.58d	0.36f
**Year × treatment**			
YIT1	3.84g	0.50c	0.17e
YIT2	4.19ef	0.53abc	0.26bcd
YIT3	4.46cd	0.54abc	0.30abc
YIT4	4.75b	0.56abc	0.30abc
YIT5	4.19ef	0.58ab	0.25cde
YIT6	4.28de	0.55abc	0.26bcd
YIT7	5.32a	0.56abc	0.36a
YIT8	5.42a	0.59a	0.36a
YIIT1	3.81g	0.50c	0.18de
YIIT2	3.98fg	0.51bc	0.24cde
YIIT3	4.50cd	0.54abc	0.31abc
YIIT4	4.61bc	0.55abc	0.31abc
YIIT5	4.14ef	0.52abc	0.25cde
YIIT6	4.25e	0.53abc	0.27bc
YIIT7	5.24a	0.56abc	0.34ab
YIIT8	5.38a	0.57abc	0.36a

Means followed by different letters (Tukey ranking) differ significantly at 5% level of significance. Treatments details are given in Table 1.

#### Plant growth analysis

**Figure 1:**
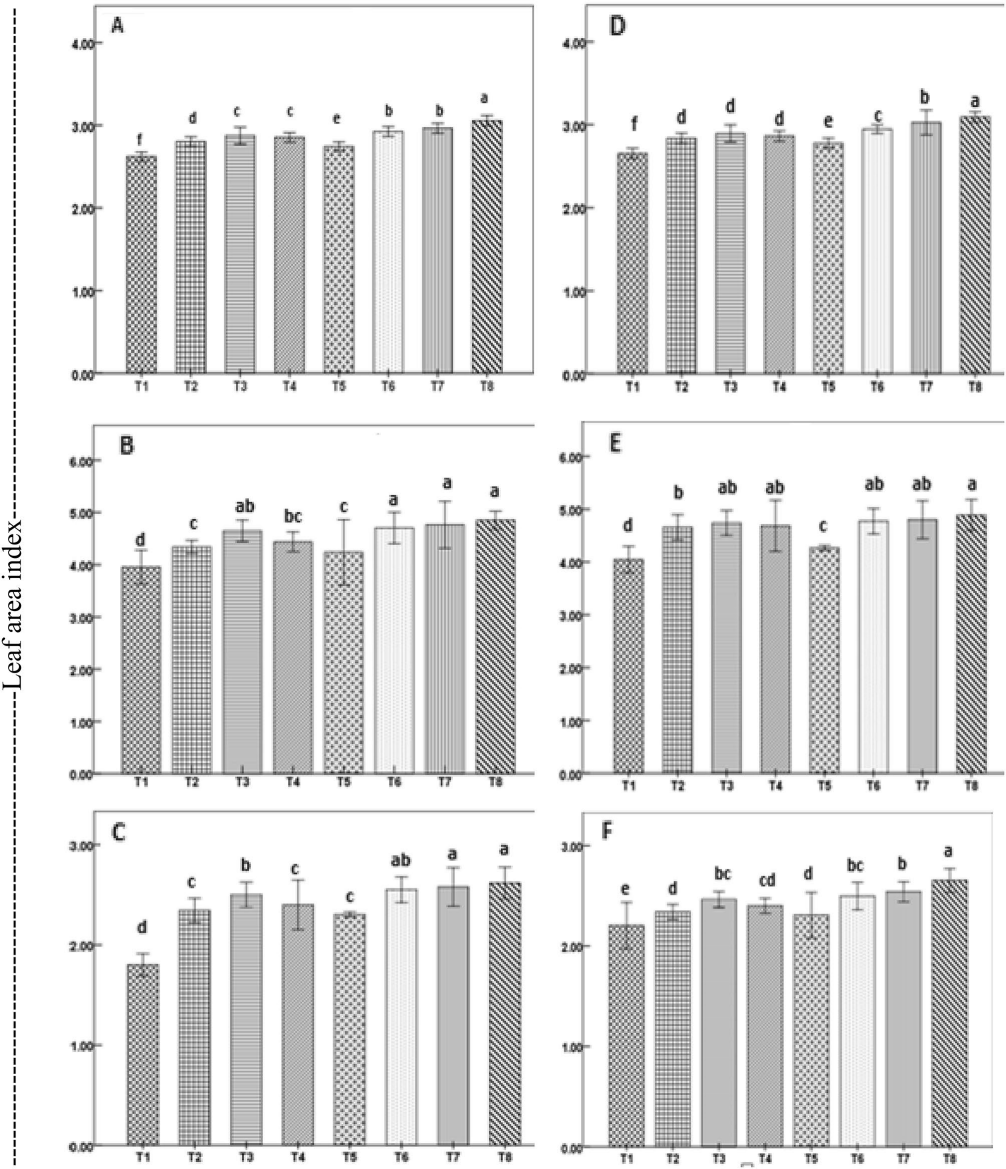
Leaf area index of direct-seeded rice plants during vegetative (**A**,**D**), reproductive (**B**,**E**), and ripening (**C**,**F**) stages as affected by seed priming treatments in a field experiment in 2019 (**A**–**C**) and 2020 (**D**–**F**). Treatments with different letters (top of bars) represent significant differences (P < 0.05) between treatments. Vertical lines with caps are ± standard error of the mean. Treatments details are given in Table 1.

All priming treatments either singly or in combination, affected chlorophyll, phenolics, and soluble protein contents of seedlings, growth behavior, nutrient uptake by plants, and crop yield at harvest (Supplementary Table S3). Year-to-year variation was also noticed in all parameters with a few exceptions (soluble proteins, CGR at the vegetative stage, and NAR at the ripening stage). However, year-treatment interactions were mostly non-significant. The impact of priming of seeds is manifest in plant LAI (Fig. 1). The highest LAI was observed with ZnO-NPs, Na-selenite, and Na-selenate combination in all stages—1.66 (early tillering), 3.50 (panicle initiation), 5.21 (early grain filling), and 3.18 (physiological maturity), with 81%, 93% 56%, and 55% higher, respectively over the control.

**Figure 2:**
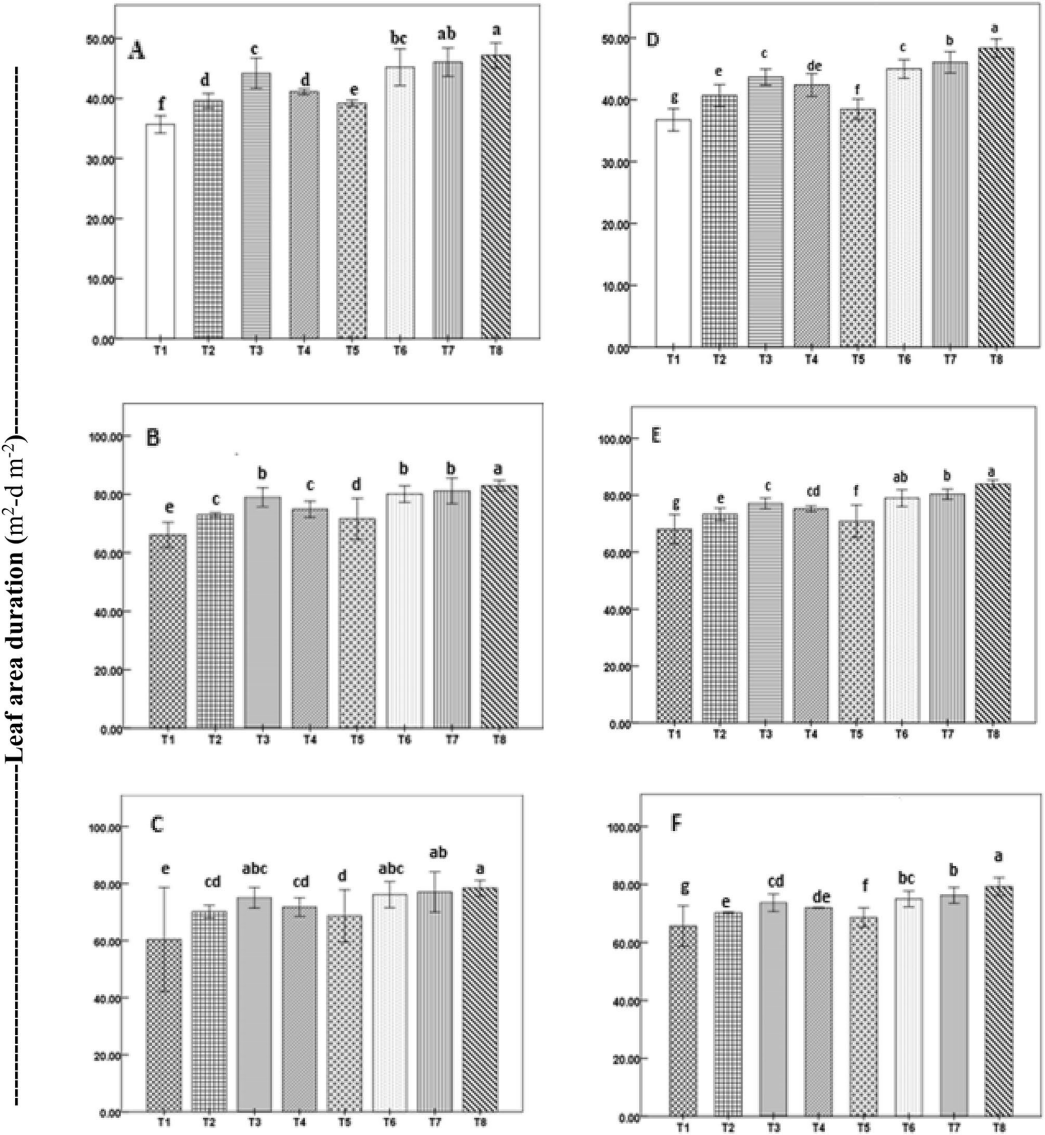
Leaf area duration of direct-seeded rice plants during vegetative (**A**,**D**), reproductive (**B**,**E**), and ripening (**C**,**F**) stages as affected by seed priming in a field experiment in 2019 (**A**–**C**) and 2020 (**D**–**F**). Treatment bars with different letters represent significant differences (P < 0.05) between means. Vertical lines with caps are ± standard error of the mean of treatments. Treatments details are given in Table 1.

**Figure 3:**
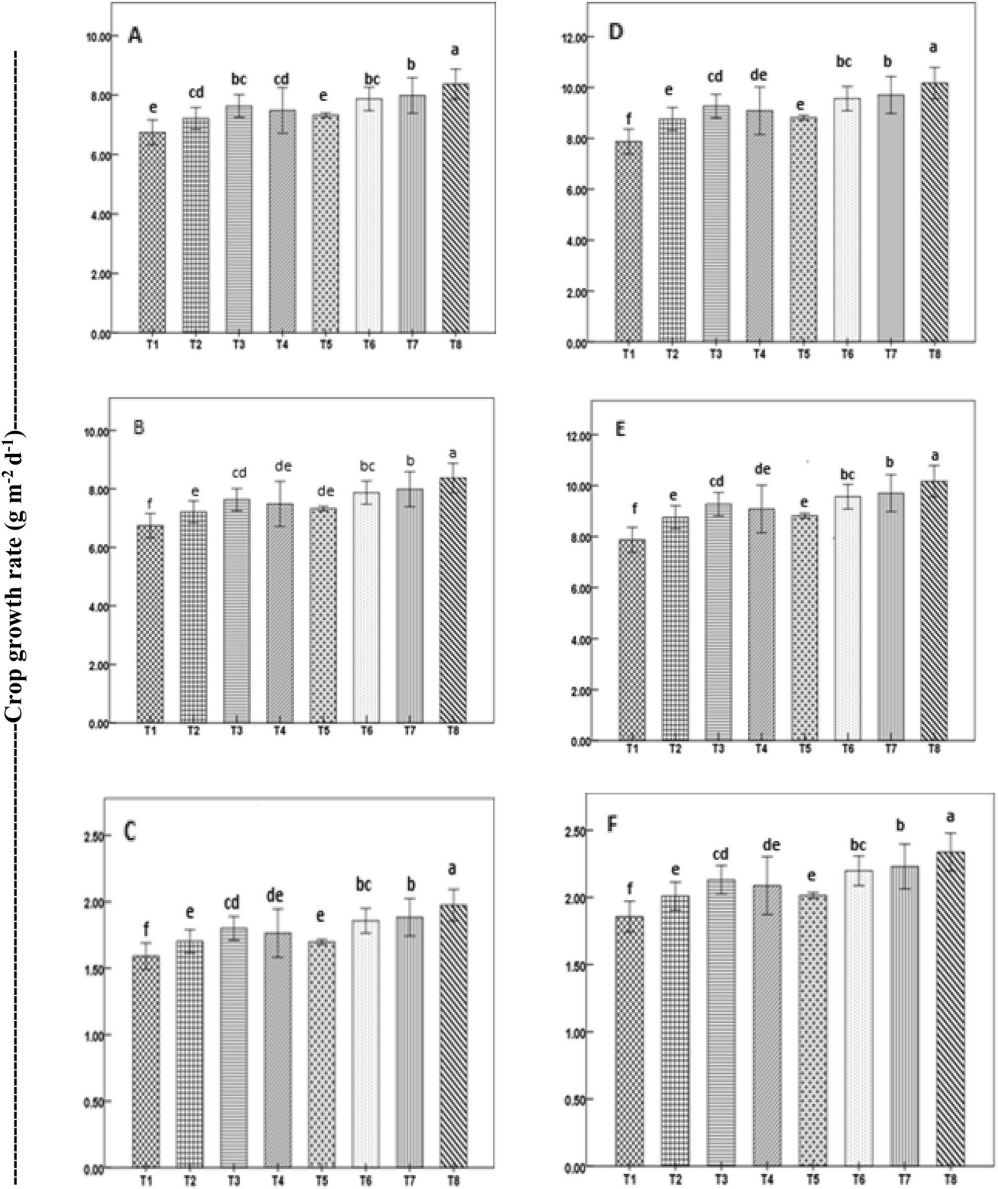
Crop growth rate of direct-seeded rice during vegetative (**A**,**D**), reproductive (**B**,**E**), and ripening (**C**,**F**) stages as affected by seed priming in a field experiment in 2019 (**A**–**C**) and 2020 (**D**–**F**). Treatment bars with different letters represent significant differences (P < 0.05) between means. Vertical lines with caps are ± standard error of the mean of treatments. Treatments details are given in Table 1.

**Figure 4:**
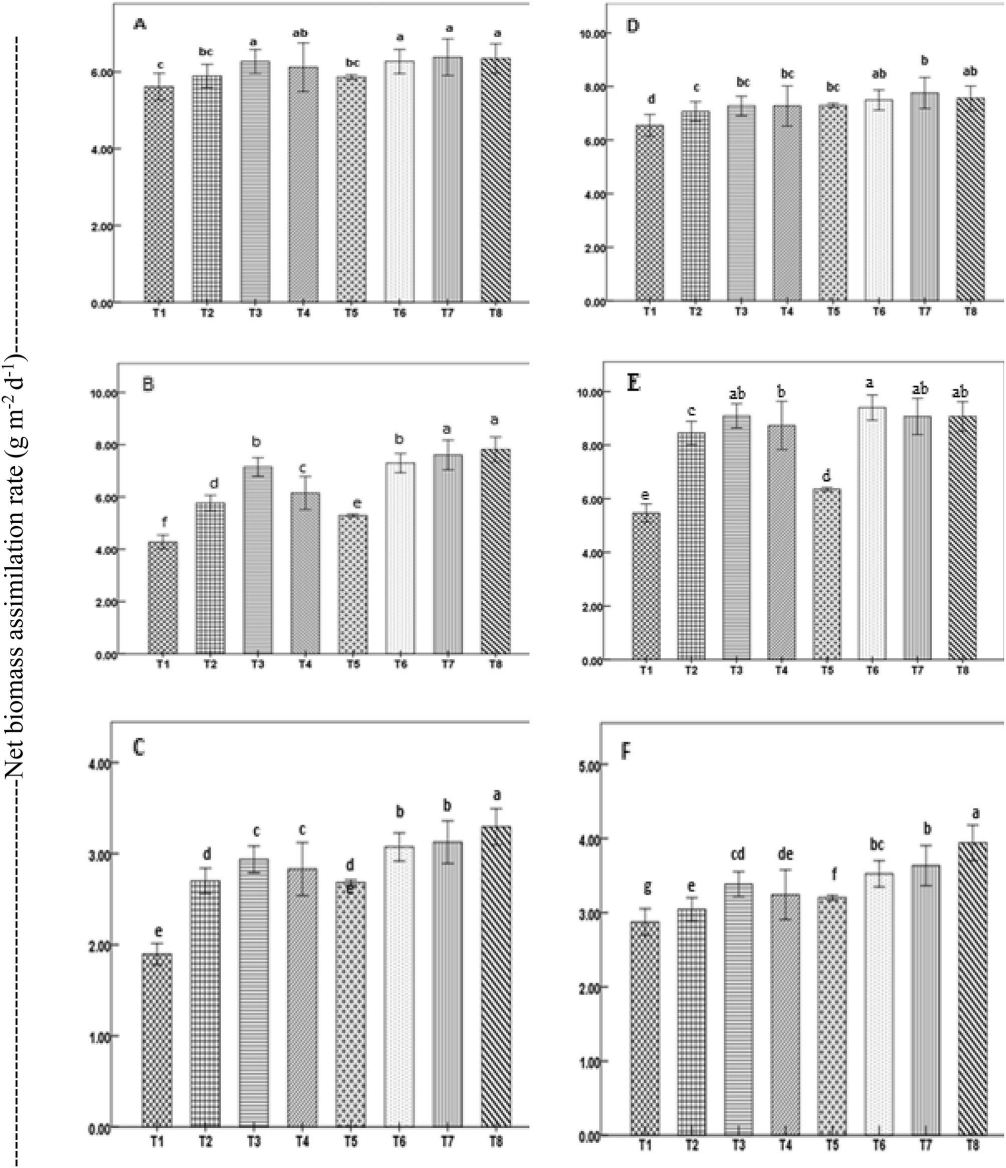
Net biomass assimilation rate by direct-seeded rice plants during vegetative (**A**,**D**), reproductive (**B**,**E**), and ripening (**C**,**F**) stages as affected by seed priming in a field experiment in 2019 (**A**–**C**) and 2020 (**D**–**F**). Treatment bars with different letters represent significant differences (P < 0.05) between means. Vertical lines with caps are ± standard error of the mean of treatments. Treatments details are given in Table 1.

Seed priming impacted other physiological parameters like LAD (Fig. 2), CGR (Fig. 3), and NAR (Fig. 4) during early tillering to panicle initiation stage (14–42 DAS), panicle initiation to completion of pollination (42–70 DAS) and entire grain filling stage (72 DAS-108 DAS).

The impact of Na-selenate on LAI and LAD was always greater than that of Na-selenite, and the combined selenite-selenate of sodium salt was even more effective. A similar trend was recorded for CGR except during 42–70 DAS where Na-selenate alone and with Na-selenite had a comparable effect. Seed priming by ZnO-NPs alone or with Na-selenite did not bring a change in either LAI or LAD, although the effect was greater with Na-selenate and was the best when combined with Na-selenite and Na-selenate. Likewise, a combination of ZnO-NPs with Na-selenite and Na-selenate together had the best priming effect on CGR. Overall, improved LAI and LAD with seed priming contributed 9–55% higher CGR and 34–109% higher NAR in comparison to control. The NAR showed a mixed trend, with all treatments behaving similarly at14-42 DAS although all three agents in combination showed less NAR compared to single ZnO-NPs. During 42–70 DAS, ZnO-NPs alone or in combination with both Na-selenite and Na-selenate recorded lower NAR, however, all treatments had a similar effect during 70–108, but higher than the control.

#### Nutrient uptake by plants

Nutrient uptake (153 kg N ha^−1^, 48.6 kg P ha^−1^, 78.7 kg K ha^−1^, 24.6 kg B ha^−1^, 321 g Zn ha^−1^, and 188 kg Si ha^−1^) was the highest with the combined use of Na-selenite, Na-selenate, and ZnO-NPs (Table 3). The efficacy of Na-selenate was greater in harnessing soil nutrients compared to either Na-selenite or ZnO-NPs.

**Table 3:** Effect of seed priming on total plant uptake (kg ha^-1^) of primary nutrients and micronutrients by direct-seeded rice in a field experimentduring 2019 (YI) and 2020 (YII).

Treatment	N	P	K	B	Zn	Si
**Year**						
YI	131o	38.4k	68.8l	21.1m	276k	162m
YII	127p	37.4l	66.7m	20.4n	266l	157n
**Treatment**						
T1	109n	28.9j	53.8k	15.8k	206j	122l
T2	119m	36.4h	64.2j	19.9i	260h	153j
T3	128l	38.4h	68.5ij	21i	275h	162j
T4	125lm	36.5h	66.9ij	10i	261h	154j
T5	118m	31.7i	67.3ij	17.4j	227i	134k
T6	137k	42.5g	69i	23.2h	304g	178i
T7	141k	43.8fg	73.6h	24.0g	313g	184hi
T8	153j	44.9f	78.7g	24.6g	321g	188h
**Year × treatment**						
YIT1	121fg	29.2e	56.6ef	16.0f	208f	124fg
YIT2	97i	36.8c	51.0f.	20.2d	264d	155d
YIT3	127efg	38.9bc	69.2cd	21.3cd	279cd	120g
YIT4	129def	37.3c	70.0cd	20.5d	268d	165bcd
YIT5	130def	32.1de	78.7a	17.7ef	231ef	158d
YIT6	132def	43.1a	63.4de	23.6ab	310ab	137ef
YIT7	136cde	44.5a	70.1bcd	24.5ab	318ab	180ab
YIT8	156a	45.6a	78.5a	25.1a	327a	187a
YIIT1	116gh	28.6e	64.2d	15.6f	204f	191a
YIIT2	122fg	36.0cd	64.2d	19.6de	256de	151de
YIIT3	129def	38.0bc	67.8cd	20.7cd	271cd	159cd
YIIT4	121fg	35.7cd	63.8d	19.5de	254de	150de
YIIT5	106hi	31.3e	55.9f	17.1f	223f	131fg
YIIT6	142bcd	41.9ab	74.7abc	22.8bc	298bc	176abc
YIIT7	146abc	43.2a	77.1ab	23.5ab	308ab	181ab
YIIT8	150ab	44.2a	78.9a	24.1ab	315ab	185a

Means followed by different letters (Tukey ranking) differ significantly at 5% level of significance. Treatments detail in Table 1.

#### Yield attributes and grain and straw yield

Seed priming improved the grain yield in DSR due to an increase in panicle density, grains per panicle, and grain-filling (Table 4). Grain yield ranged from 3.91t ha^−1^ in the second year with hydropriming treatment to 6.15 t ha^−1^ in the first year with combined application of ZnO-NPs, Na-selenite, and Na-selenate. Yields were significantly lower in YII than in Y1. Straw yield followed a similar pattern. Application of Na-selenite and Na-selenate with ZnO-NPs registered a 25.9% higher grain yield over the control, compared to 11–13% increase with application of ZnO-NPs with either Na-selenite or Na-selenate. Yield gains in treatment with the combination of all three priming agents could be ascribed to 12.0, 47.1, and 27.8% increase in panicles, filled grains per panicle, and grain filling percentage respectively over the control. Combined application of ZnO-NPS and Na-selenite or ZnO-NPS and Na-selenate also resulted in 7.6–8.7, 32.8–32.9, 22.2–23.6% higher panicles, filled grains per panicle, and grain-filling efficiency respectively over control. However, sole use of Na-selenite, Na-selenate, and ZnO-NPs also contributed 5.5, 6.2, and 5.0% higher grain yield over hydro priming.

**Table 4:** Effect of seed priming on yield and yield components of direct-seeded rice in a field experiment during 2019 (YI) and 2020 (YII).

Treatment	Panicles m^−2^	Filled grain panicle^−1^	Grain filling percentage	Test weight (g)	Grain yield (t ha^−1^)	Straw yield (t ha^−1^)	Harvest index (%)
**Year**							
YI	174j	114k	85g	20.24	4.53k	5.81j	43.8i
YII	165i	102j	86g	20.22	4.22j	5.57i	43.1i
**Treatment**							
T1	184h	85i	72f	20.10	4.01i	5.70g	41.3h
T2	188gh	105h	84e	20.40	4.23h	5.68g	42.6gh
T3	192g	110fg	88e	20.10	4.26h	5.66g	42.9fgh
T4	192g	110fg	87e	20.20	4.32h	5.68g	43.2fgh
T5	188gh	103g	83e	20.50	4.21h	5.62gh	42.8gh
T6	200ef	113f	88e	20.10	4.53g	5.80fg	43.9efg
T7	198f	113f	89e	20.17	4.45g	5.46h	44.9ef
T8	206e	125e	92d	20.28	5.05f	5.96f	45.9e
**Year × treatment**							
YIT1	189d	88d	68c	20.13	4.10e	5.70e	40.8d
YIT2	191c	112b	85b	20.45	4.37cd	5.81c	42.4bcd
YIT3	196c	118b	90a	20.11	4.41c	5.75c	42.4bcd
YIT4	198bc	109bc	85b	20.17	4.49c	5.91b	43.2abcd
YIT5	194c	115b	84b	20.49	4.36c	5.66d	42.1bcd
YIT6	205ab	119b	91a	20.04	4.70b	5.92b	43.5abcd
YIT7	202ab	117b	88b	20.07	4.62b	5.59b	44.5abc
YIT8	209a	130a	91a	20.45	5.22a	6.15a	45.8a
YIIT1	179d	82d	76c	20.07	3.91e	5.68c	41.8cd
YIIT2	185c	98c	84b	20.35	4.08cd	5.55b	42.9bcd
YIIT3	188c	101bc	86b	20.09	4.10c	5.57b	43.4abcd
YIIT4	186c	112b	88b	20.23	4.14c	5.44b	43.2abcd
YIIT5	182c	90c	82b	20.51	4.05d	5.57b	43.5abcd
YIIT6	195b	106b	85b	20.16	4.36b	5.67b	44.3abc
YIIT7	194b	109b	90a	20.27	4.27b	5.32b	45.3ab
YIIT8	203a	121a	93a	20.11	4.87a	5.77a	45.9a

Within a column, means followed by the same letter are not different at the 0.05 level of probability. Treatments detail in Table 1.

## Discussion

Seed priming by ZnO-NPs, Na-selenite, and Na-selenate either singly or in combinations, facilitated speedy germination and early vigour of seedlings in the DSR. Priming reduced the time to start emergence as well as the time to reach 50% germination. Primed seeds have shown low MET, a measure of the rate and time-spread of germination^84^, and enhanced seedling emergence and vigour. There were increases in enzymatic antioxidant functions in seeds necessary to reduce the production of degenerative radicals and therefore, seeds would likely be protected from damage due to environmental stresses during the growing stages. Consequently, seedling length (root and shoot) largely increased at18 DAS (Supplementary Figure S3). All enzymatic activities were positively correlated with seed vigour indices and the seedling growth following seed priming (Supplementary Figure S4). Na-selenate had a clear advantage over Na-selenite when combined with ZnO-NPs. However, the combination of all three priming agents had the maximum impact.

Early vigour of seeds and seedlings is the most desirable trait in field crops to enhance water and nutrient uptake by plants and to provide competitiveness against biotic and abiotic stresses^7,85^. Due to the chemical priming of seeds, plants used soil nutrients (N, P, K, B, Zn, Si) more efficiently resulting in a higher density of grains per panicle and finally leading to a higher yield in DSR compared to grain imbibition with pure water. Na-selenate improved rice seedling emergence and growth, and soluble carbohydrate and protein contents in plants compared to hydropriming^56,86^. The ZnO-NPs had similar positive effects on seed germination, seedling growth, and dry biomass in rice^87^ and wheat^88^. The internalized ZnO-NPsin primed seeds were several times higher compared to hydropriming which could induce gene expression favouring metabolic activities that enhanced germination performance and seedling vigour^87,89^. The success with ZnO-NPs may encourage future research on other micronutrients as priming agents^87^.

Chlorophyll is associated with photosynthetic activity, and hence it is an indicator of vegetative growth and vigour of a plant. Phenolics are secondary metabolites, which promote the adaptation capability of plants during stress. Soluble proteins have diverse roles in promoting growth including osmoregulation in plants under adverse growth environments^90^. A combination of ZnO-NPs, Na-selenite and Na-selenate priming had the best impact on chlorophyll, phenol and soluble protein contents in rice seedlings, resulting in higher LAI and LAD, and CGR. A combination of Na-selenite and Na-selenate was more effective in promoting the growth of plants (higher LAI, LAD, and CGR) compared to when these were used singly or even when seeds were primed by nano-zinc formulation only. However, the combination of both Na-selenite and Na-selenate with nano-zinc induced the largest changes in growth parameters. These led to enhanced yield-contributing factors such as increased grain-filling efficiency, test weight, and harvest index. The highest DSR yield was, however, obtained with a combination of the three seed priming materials.

Our results established that combinations of Na-selenite, Na-selenate, and ZnO-NPs were potential seed invigoration techniques, and the best results were obtained with all three priming agents in combination. Though responses differed between years, positive impacts on DSR in the field were established. Seeds with high vigour are a proxy of sustainable productivity, especially under adverse conditions, although suitable field studies are lacking. Uniform seedling emergence, and improved crop stand and establishment are major challenges for successful DSR cultivation^18^. DSR often fails to emerge uniformly due to uneven land preparation leading to imprecise water management in both irrigated and rainfed conditions in both uplands and lowlands, and therefore weed infestations take place heavily^91^. In particular, seeds fail to germinate due to low oxygen availability in rainfed lowlands owing again to poor water management^92^. Priming of DSR seeds can give necessary vigour to the plants to sustain better under unfavorable growing conditions. The modified traits in seeds effectively translate into early seedling vigour and agility in growing plants, which is perfectly tuned with increased productivity in the field-grown rice. This small intervention through seed priming will support the rice growers to embrace DSR as a replacement of water- and labour-intensive PTR. Farmers will also get an opportunity to widen the window of sowing of wheat following rice in large tracts of rice–wheat rotation in the Indo-Gangetic Plains of South Asia^12^.

## Conclusion

Both Zn and Se have found their effectiveness in seed-priming. It was observed that ZnO-NPs with both Na-selenite and Na-selenate forms were synergistic in their action, and provided further benefits compared to their single applications. The impact was evaluated in DSR which is often exposed to adverse growing conditions and therefore, not realizing its full potential productivity. Priming of seeds with ZnO-NPs, Na-selenite, and Na-selenate combination resulted in an early seedling emergence due to increased seed vigour, and improved plant growth and productivity in DSR in the field. This will allow sustainable intensification in the vast rice-growing areas in South Asia in general and the rice–wheat system of the Indo-Gangetic Plains of South Asia in particular.

## Supplementary Information

Supplementary Information.
