# Supplementary material for: Seed priming with selenium and zinc nanoparticles modifies germination, growth, and yield of direct-seeded rice (Oryza sativa L.)
Source: Sci Rep. 2022 May 2;12:7103. doi: 10.1038/s41598-022-11307-4 (PMC9061837; doi:10.1038/s41598-022-11307-4)
Supplement: Supplementary file 1 — Supplementary Information. [file 41598_2022_11307_MOESM1_ESM.docx]

**Supplementary File**

**Supplementary Table S1** Results of the analysis of variance (two-way ANOVA with interaction) of seed parameters

| Variables | Sources of variation | Df | Sum of squares | F ratio | Prob |
| --- | --- | --- | --- | --- | --- |
|  |  |  |  |  |  |
| Time to start emergence | Year | 1 | 0.750000 | 3.6000 | 0.0668 |
|  | Treatment | 7 | 11.250000 | 7.7143 | <.0001* |
|  | Year x Treatment | 7 | 1.250000 | 0.8571 | 0.5499 |
| Time taken to reach to 50% emergence | Year | 1 | 0.299252 | 4.7383 | 0.0370* |
|  | Treatment | 7 | 11.529531 | 26.0794 | <.0001* |
|  | Year x Treatment | 7 | 1.246465 | 2.8195 | 0.0209* |
| Mean emergence time | Year | 1 | 0.34199 | 0.2662 | 0.6094 |
|  | Treatment | 7 | 142.51018 | 15.8494 | <.0001* |
|  | Year x Treatment | 7 | 22.48483 | 2.5007 | 0.0361* |
| Emergence index | Year | 1 | 10.85263 | 7.0387 | 0.0123* |
|  | Treatment | 7 | 297.35099 | 27.5503 | <.0001* |
|  | Year x Treatment | 7 | 19.09522 | 1.7692 | 0.1282 |
| Vigour index | Year | 1 | 340969.9 | 107.0557 | <.0001* |
|  | Treatment | 7 | 4981929.0 | 223.4565 | <.0001* |
|  | Year x Treatment | 7 | 19137.7 | 0.8584 | 0.5490 |

*Biochemical analysis of seeds*

Primed seeds (5 g) were washed thrice with deionized water to remove any salt imposition, and then soaked in 50 mL of deionized water at 25 °C. The electrical conductivity (EC) of seed leachates was measured after 0.5, 1, 1.5, 2, 2.5, 6, 12 and 24 h of soaking with a digital conductivity meter (HI-9811, Hannah Instruments, USA). Lipid peroxidation of treated and non-treated rice seeds was determined as malondialdehyde (MDA) content using the thiobarbituric acid method ^82^. The activity of α-amylase of ground and powdered rice seeds (1 g) was measured as per procedure given by Lee and Kim (2000). The rate at which maltose is liberated from starch was measured by its ability to reduce 3,5-dinitrosalicyclic acid. Total soluble sugars and soluble protein contents were quantified following the methods given by Dubois et al. (1956) and Bradford (1976), respectively. Superoxide dismutase (SOD) activity was determined as described by Giannopolitis and Ries (1977). One unit of SOD activity was defined as the amount of enzyme inhibiting the photochemical reduction of nitro blue tetrazolium chloride by the half min^-1^ at 560 nm. Catalase (CAT) activity based on the consumption of H_2_O_2_ was determined based on Dhindsa et al. (1981). The consumption of H_2_O_2_ was observed at 240 nm, and one unit of CAT was defined as the amount of enzyme required to oxidize 1 μmol H_2_O_2_ min^−1^. Peroxidase (POD) activity was recorded as described by Egley et al. (1983). An increase in absorbance due to guaiacol oxidation was measured at 470 nm. One unit of enzyme activity was defined as the amount of enzyme required to oxidize 1 μmol guaiacol min^−1^. Glutathione peroxidase (GPX) activity based on conversion of reduced glutathione to oxidized form following oxidation of NADH at 340 nm was determined ^88^. One unit of enzyme activity was defined as the quantity of enzyme required to catalyze the oxidation by H_2_O_2_ of 1 μmol reduced glutathione to oxidized form at pH 7.0 and 25 °C.

With advancement of time, EC of leachate from primed rice seeds diminished more over the control (**Supplementary Figure 2**). A minimum of leachate conductivity was recorded in the combination of all three priming agents, although Se-primed seeds had lower EC of leachate compared to ZnO-NPs alone. Selenate (7.87 nmol g^-1^) was more effective in reducing MDA content than either selenite (8.17 nmol g^-1^) or nano zinc (8.31 nmol g^-1^) (**Supplementary Table 2**). Selenate was further effective in combination with selenite (7.45 nmol g^-1^ of MDA) or ZnO-NPs (7.46 nmol g^-1^) or with both (7.36 nmol g^-1^). Although better efficacy was observed with respect to increase in α-amylase activity of selenate (8.79 units) over that of selenite (7.88 units) or ZnO-NPs (7.88 units), combined application of all three priming agents recorded the highest α-amylase activity (9.53 units). Soluble sugar content, a major product of α-amylase activity, was also the highest in seed leachate with combination of all three (18.07 mg g^-1^) followed by selenate + ZnO-NPS (17.79 mg g^-1^), selenite + selenate (17.17 mg g^-1^), only selenite (16.35 mg g^-1^) and only ZnO-NPs (16.49 nm g^-1^). Combined application of all three priming agents also enhanced the activity of antioxidant enzymes SOD (991 units g^-1^ protein), POX (3.37 units g^-1^ protein), CAT (2.59 units g^-1^ protein) and GPX (324 µmol min^-1^ g^-1^ protein) compared to the control.

**Supplementary Table S2** Effect of priming on seed biochemical attributes in DSR

| Treatments | MDA | α-Amylase  activity | Total soluble sugars | SOD | POX | CAT | GPX |
| --- | --- | --- | --- | --- | --- | --- | --- |
|  | n mol g^−1^ seed) | units^a^ | mg g^−1^ seed | units g^−1^ protein | ------μmol min^−1^ g^−1^ protein------ | | |
| T_1_ | 8.61a | 7.19h | 15.84h | 640h | 1.30h | 1.24h | 174h |
| T_2_ | 8.17b | 7.88g | 16.35g | 790g | 2.88f | 1.86g | 196g |
| T_3_ | 7.87c | 8.79d | 17.17d | 831d | 2.97d | 2.27d | 251d |
| T_4_ | 7.45d | 8.95c | 17.31c | 838c | 3.03c | 2.35c | 309c |
| T_5_ | 8.31b | 8.41f | 16.49f | 807f | 2.86g | 2.07f | 210f |
| T_6_ | 8.18b | 8.64e | 16.80e | 821e | 2.92e | 2.13e | 234e |
| T_7_ | 7.46d | 9.24b | 17.79b | 973b | 3.28b | 2.41b | 320b |
| T_8_ | 7.36d | 9.53a | 18.07a | 991a | 3.37a | 2.59a | 334a |
| HSD p ≤ 0.05 | 0.24 | 0.20 | 0.19 | 24 | 0.06 | 0.14 | 10 |

^a^One unit refers to the amount of enzyme which released 1 μmol of maltose by 1 mL original enzyme solution in 1 min.

Means not sharing a letter in common differ significantly at 5% probability level by HSD Tukey’s test.

For treatments details, refer to the Main Text (Table 1)

**Supplementary Table S3** Results of the analysis of variance (two-way ANOVA with interaction) test of the field experimentation

| Parameters | Sources of variation | df | Sum of squares | F ratio | Prob |
| --- | --- | --- | --- | --- | --- |
| Total chlorophyll | Year | 1 | 0.060224 | 11.9605 | 0.0016* |
|  | Treatment | 7 | 13.471943 | 382.2212 | <.0001* |
|  | Year x Treatment | 7 | 0.057524 | 1.6320 | 0.1622 |
| Total phenolics | Year | 1 | 0.00310556 | 4.5914 | 0.0398* |
|  | Treatment | 7 | 0.02828314 | 5.9736 | 0.0002* |
|  | Year x Treatment | 7 | 0.00452202 | 0.9551 | 0.4796 |
| Soluble protein | Year | 1 | 6.24e-12 | 0.0000 | 0.9999 |
|  | Treatment | 7 | 0.15773311 | 29.0520 | <.0001* |
|  | Year x Treatment | 7 | 0.00131876 | 0.2429 | 0.9709 |
| N | Year | 1 | 208.9003 | 10.8107 | 0.0025* |
|  | Treatment | 7 | 8480.7262 | 62.6975 | <.0001* |
|  | Year x Treatment | 7 | 2038.1335 | 15.0678 | <.0001* |
| P | Year | 1 | 14.0359 | 8.4033 | 0.0067* |
|  | Treatment | 7 | 1373.0465 | 117.4348 | <.0001* |
|  | Year x Treatment | 7 | 1.1087 | 0.0948 | 0.9983 |
| K | Year | 1 | 55.9556 | 10.5443 | 0.0027* |
|  | Treatment | 7 | 2188.2826 | 58.9089 | <.0001* |
|  | Year x Treatment | 7 | 1098.2948 | 29.5663 | <.0001* |
| B | Year | 1 | 6.58412 | 13.1523 | 0.0010* |
|  | Treatment | 7 | 413.31505 | 117.9474 | <.0001* |
|  | Year x Treatment | 7 | 0.51862 | 0.1480 | 0.9931 |
| Zn | Year | 1 | 1088.591 | 12.7591 | 0.0011* |
|  | Treatment | 7 | 70520.778 | 118.0793 | <.0001* |
|  | Year x Treatment | 7 | 90.171 | 0.1510 | 0.9927 |
| Si | Year | 1 | 367.141 | 12.4498 | 0.0013* |
|  | Treatment | 7 | 23365.702 | 113.1909 | <.0001* |
|  | Year x Treatment | 7 | 17.933 | 0.0869 | 0.9987 |
| LAI (Vegetative stage) | Year | 1 | 1.932019 | 63.8552 | <.0001* |
|  | Treatment | 7 | 13.039931 | 61.5690 | <.0001* |
|  | Year x Treatment | 7 | 1.126331 | 5.3181 | 0.0004* |
| LAI (Reproductive stage) | Year | 1 | 13.409102 | 348.2130 | <.0001* |
|  | Treatment | 7 | 15.896065 | 58.9708 | <.0001* |
|  | Year x Treatment | 7 | 1.006915 | 3.7354 | 0.0046* |
| LAI (Ripening stage) | Year | 1 | 0.779811 | 32.3316 | <.0001* |
|  | Treatment | 7 | 14.214487 | 84.1920 | <.0001* |
|  | Year x Treatment | 7 | 0.338308 | 2.0038 | 0.0855 |
| LAD (Vegetative stage) | Year | 1 | 349.2635 | 51.9252 | <.0001* |
|  | Treatment | 7 | 5154.0830 | 109.4658 | <.0001* |
|  | Year x Treatment | 7 | 184.3468 | 3.9153 | 0.0034* |
| LAD (Reproductive stage) | Year | 1 | 5002.083 | 551.2555 | <.0001* |
|  | Treatment | 7 | 11063.364 | 174.1771 | <.0001* |
|  | Year x Treatment | 7 | 460.058 | 7.2430 | <.0001* |
| LAD (Ripening stage) | Year | 1 | 1513.432 | 118.7769 | <.0001* |
|  | Treatment | 7 | 11725.229 | 131.4597 | <.0001* |
|  | Year x Treatment | 7 | 304.565 | 3.4147 | 0.0077* |
| Crop growth rate (Vegetative stage) | Year | 1 | 0.040750 | 1.5227 | 0.2262 |
|  | Treatment | 7 | 24.762983 | 132.1863 | <.0001* |
|  | Year x Treatment | 7 | 1.079833 | 5.7642 | 0.0002* |
| Crop growth rate (Reproductive stage) | Year | 1 | 7.736962 | 67.1683 | <.0001* |
|  | Treatment | 7 | 46.781458 | 58.0189 | <.0001* |
|  | Year x Treatment | 7 | 7.214922 | 8.9480 | <.0001* |
| Crop growth rate (Ripening stage) | Year | 1 | 5.250039 | 66.8017 | <.0001* |
|  | Treatment | 7 | 34.913538 | 63.4630 | <.0001* |
|  | Year x Treatment | 7 | 3.639732 | 6.6160 | <.0001* |
| Net assimilation rate (Vegetative stage) | Year | 1 | 0.8752610 | 32.3202 | <.0001* |
|  | Treatment | 7 | 1.2093813 | 6.3797 | <.0001* |
|  | Year x Treatment | 7 | 0.8505056 | 4.4866 | 0.0014* |
| Net assimilation rate (Reproductive stage) | Year | 1 | 1.1187662 | 48.7605 | <.0001* |
|  | Treatment | 7 | 1.1887455 | 7.4015 | <.0001* |
|  | Year x Treatment | 7 | 0.4765625 | 2.9672 | 0.0163* |
| Net assimilation rate (Ripening stage) | Year | 1 | 0.00997034 | 0.9205 | 0.3445 |
|  | Treatment | 7 | 0.28774566 | 3.7951 | 0.0042* |
|  | Year x Treatment | 7 | 0.40693454 | 5.3671 | 0.0004* |
| Panicles m^-2^ | Year | 1 | 240.139 | 33.7857 | <.0001* |
|  | Treatment | 7 | 40789.948 | 819.8331 | <.0001* |
|  | Year x Treatment | 7 | 399.363 | 8.0268 | <.0001* |
| Filled grain panicle^-1^ | Year | 1 | 410.0981 | 112.9191 | <.0001* |
|  | Treatment | 7 | 2718.3090 | 106.9253 | <.0001* |
|  | Year x Treatment | 7 | 5.7049 | 0.2244 | 0.9766 |
| Grain-filling percentage | Year | 1 | 60.7500 | 10.0110 | 0.0034* |
|  | Treatment | 7 | 3461.2500 | 81.4832 | <.0001* |
|  | Year x Treatment | 7 | 17.2500 | 0.4061 | 0.8914 |
| Test weight (g) | Year | 1 | 0.00275415 | 0.0014 | 0.9700 |
|  | Treatment | 7 | 0.95390078 | 0.0713 | 0.9993 |
|  | Year x Treatment | 7 | 0.27424650 | 0.0205 | 1.0000 |
| Grain yield (t ha^-1^) | Year | 1 | 0.193761 | 8.2533 | 0.0072* |
|  | Treatment | 7 | 29.133453 | 177.2790 | <.0001* |
|  | Year x Treatment | 7 | 0.018018 | 0.1096 | 0.9973 |
| Straw yield (t ha^-1^) | Year | 1 | 1.278187 | 31.6232 | <.0001* |
|  | Treatment | 7 | 24.561631 | 86.8102 | <.0001* |
|  | Year x Treatment | 7 | 0.053628 | 0.1895 | 0.9856 |
| Harvest index (%) | Year | 1 | 6.275314 | 5.3625 | 0.0271* |
|  | Treatment | 7 | 84.405704 | 10.3040 | <.0001* |
|  | Year x Treatment | 7 | 2.600347 | 0.3174 | 0.9406 |

| ^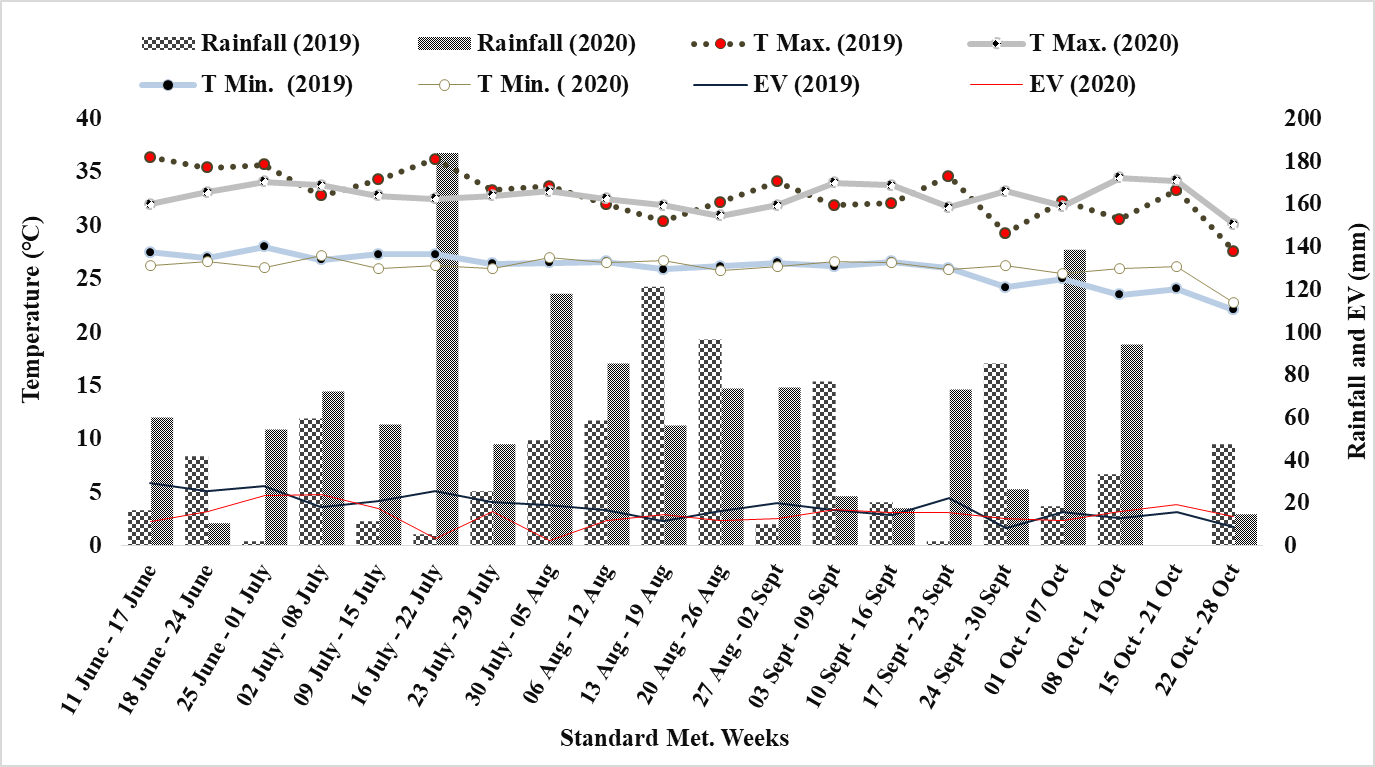^ |
| --- |
| (a). |
| ^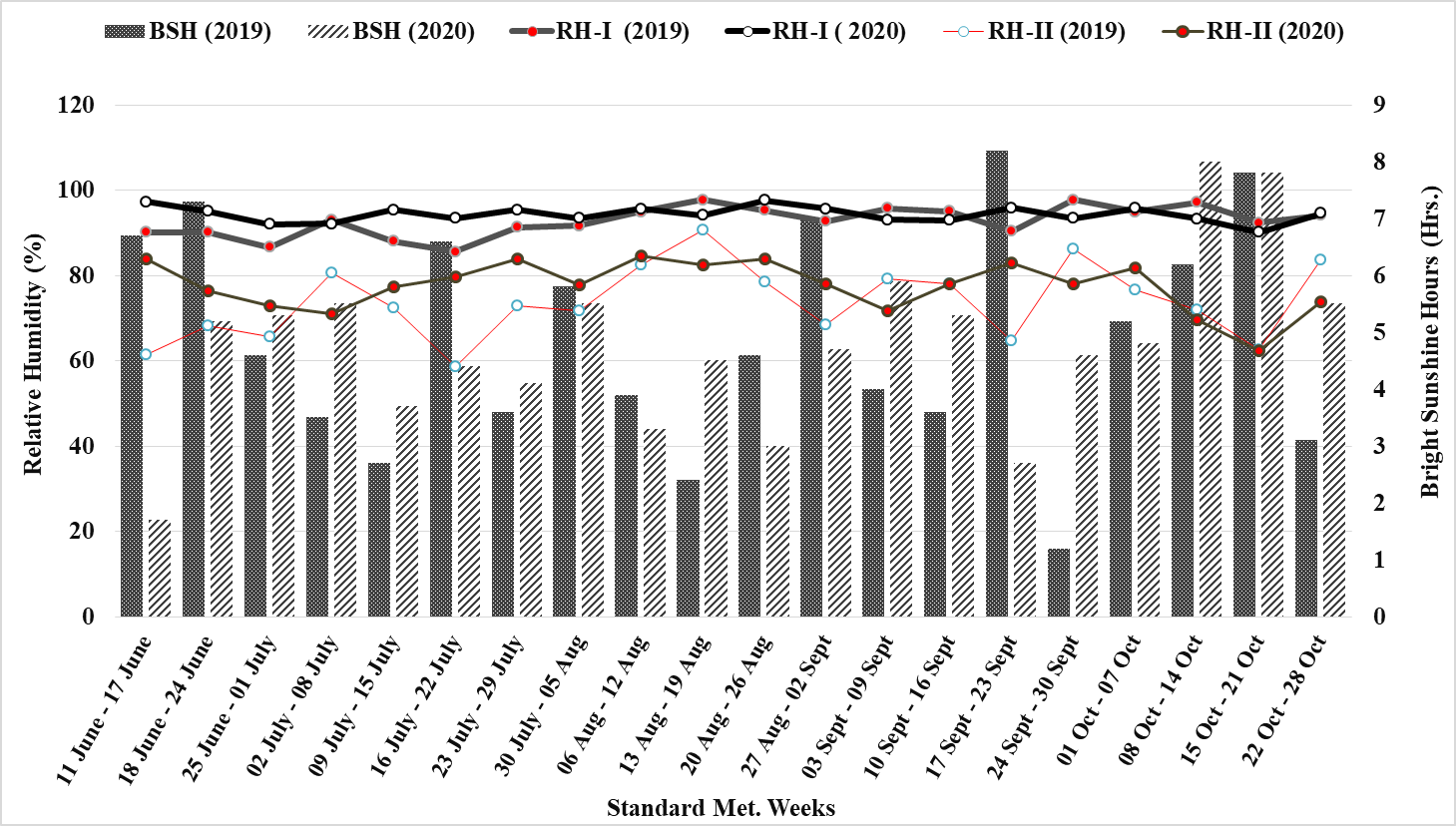^ |
| (b). |
| **Supplementary Figure S1** Weekly total rainfall, average maximum and minimum temperatures (T max and T min) and pan evaporation (a); relative humidity (morning, RH-I and evening, RH-II) and bright sunshine hours (BSH) during two years of experimentation, 2019 and 2020. |

| 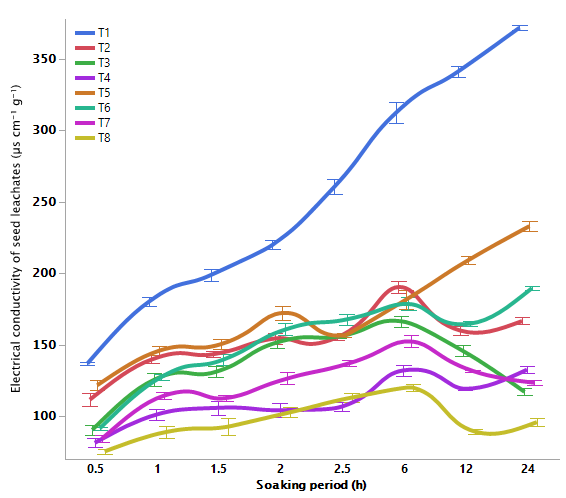 |
| --- |
| **Supplementary Figure S2** Effect of seed priming on electrical conductivity of seed leachates (µs cm^−1^ g^−1^) in direct-seeded rice as a function of soaking time. Means followed by different letters (Tukey ranking) differ significantly at 5% level of significance.  Treatments detail in Table 1 (Main Text). |

*Seedling growth*

Root and shoot lengths were measured on 18 days after sowing (DAS) from each treatment. For these, five randomly selected seedlings were cut aboveground and oven-dried at 70 °C for 48 h to get the dry biomass of root and shoot, and both the components were summed up to record the total seedling biomass.

Shoot length of seedlings increased through priming (Supplementary Figure S3). Priming with selenite and selenate combination recorded higher shoot length compared to their single application. Inclusion of ZnO-NPs with either of selenite and selenate did not show further change, but recorded the maximum shoot length (9.6cm) when combined with both of these. Priming with selenate increased root length compared to the control, but was comparable with selenite. Impact of ZnO-NPs in combination with either selenite and selenate was also similar. It was all-three-combination (ZnO-NPs+selenite+selenate) that produced the maximum root length (12.9cm) of rice seedlings.

| ^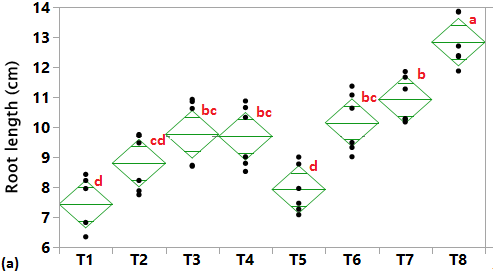^ |
| --- |
| 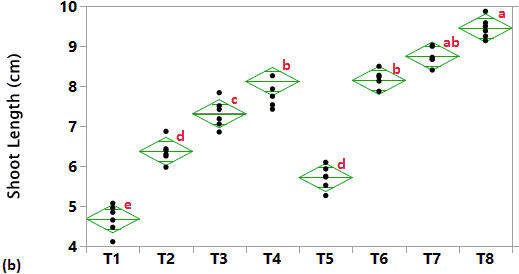 |
| **Supplementary Figure S3** Effect of seed priming with selenium and ZnO-NPs on seedling length (a) and dry weight (b) of rice in a pot experiment.  Means followed by different letters (Tukey ranking) differ significantly at 5% level of significance.  Treatments details are in Table 1 of Main Text. |

*Correlation between seed germination, biochemical attributes of seeds and seedling growth*

Correlation among the parameters characterizing seed gemination (MET and SVI), seed biochemistry (MDA, α-amylase, TSS, SOD, POX, CAT and GPX), and seedling growth (root and shoot length and seed dry biomass) are presented in **Supplementary Figure S4**. The MDA content in seeds was negatively correlated with SVI (r = - 0.77) while α-amylase activity was positively correlated. Other germination parameter MET is favouably related to α-amylase r = 0.82). MET (r = 0.77) and SVI (r = 0.90) were also positively correlated with soluble sugars (TSS), and so is the seedling dry biomass with α-amylase (r=0.80) and soluble sugars (r=0.81). All antioxidant enzymes were strongly in favour of increased shoot and root length (r between 0.63 to 0.85) and seedling dry biomass (r 0.63 to 0.82). It was also evident from the correlogram that the seedling growth was strongly related to the seed germination.

|  |
| --- |
| **Supplementary Figure S4** Correlogram between parameters characterizing seed germination (TSE=Time to start of emergence; MET mean emergence time; SVI Seedling vigour index), seed biochemistry (MDA mean malondialdehyde, SOD superoxide dismutase, POX peroxidase, CAT catalase, GPX glutathione peroxidase) and seedling growth (SL shoot length, RL root length, SDB seedling dry biomass) following seed priming in DSR |
